# Prevalence of SARS-CoV-2 infection and impact of vaccination in dialysis patients over two years of the pandemic

**DOI:** 10.1007/s40620-023-01754-1

**Published:** 2023-09-13

**Authors:** Paolo Hitz, Alberto Pagnamenta, Laura Pertusini, Tatiana Terrot, Yves Franzosi, Jessica Bassi, Chiara Silacci-Fregni, Valeria Gaia, Gladys Martinetti, Franco Keller, Lorenzo Berwert, Valentina Forni Ogna, Soraya Lavorato-Hadjeres, Davide Giunzioni, Andrea D’Ermo, Alan Valnegri, Paolo Ferrari, Davide Corti, Alessandro Ceschi, Pietro Cippà, Luca Piccoli, Olivier Giannini

**Affiliations:** 1 Faculty of Biomedical Sciences, Università della Svizzera italiana, 6900 Lugano, Switzerland; 2 Clinical Trial Unit, Ente Ospedaliero Cantonale, 6900 Lugano, Switzerland; 3 Department of Intensive Care, Ente Ospedaliero Cantonale, 6900 Lugano, Switzerland; 4 Division of Pneumology, University Hospital of Geneva, 1200 Geneva, Switzerland; 5 Division of Nephrology, Ente Ospedaliero Cantonale, 6900 Lugano, Switzerland; 6 Humabs Biomed SA, a subsidiary of Vir Biotechnology, 6500 Bellinzona, Switzerland; 7 Department of Laboratory Medicine, Ente Ospedaliero Cantonale, 6500 Bellinzona, Switzerland; 8 Department of Medicine, Ente Ospedaliero Cantonale, 6500 Bellinzona, Switzerland; 9 Process Organization and Information Service, Ente Ospedaliero Cantonale, 6500 Bellinzona, Switzerland; 10 Clinical School, University of New South Wales, Sydney, 2052 Australia; 11 Division of Clinical Pharmacology and Toxicology, Institute of Pharmacological Sciences of Southern Switzerland, Ente Ospedaliero Cantonale, 6900 Lugano, Switzerland; 12 Department of Clinical Pharmacology and Toxicology, University Hospital Zurich, 8091 Zurich, Switzerland; 13 Ospedale Regionale di Mendrisio, Via Turconi 23, CH-6850 Mendrisio, Switzerland

Dialysis patients were found to have a greater risk of severe Coronavirus disease 2019 (COVID-19) course, but epidemiological data and clinical outcome during the pandemic in this population have been largely under-reported and often discrepant. In this study we analyzed prevalence of SARS-CoV-2 infection and the impact of vaccination on clinical outcome in dialysis patients treated in the public multisite hospital of Ticino region in Southern Switzerland during the first two years of the COVID-19 pandemic (Fig. 1 and Supplementary Information).

**Fig. 1:**
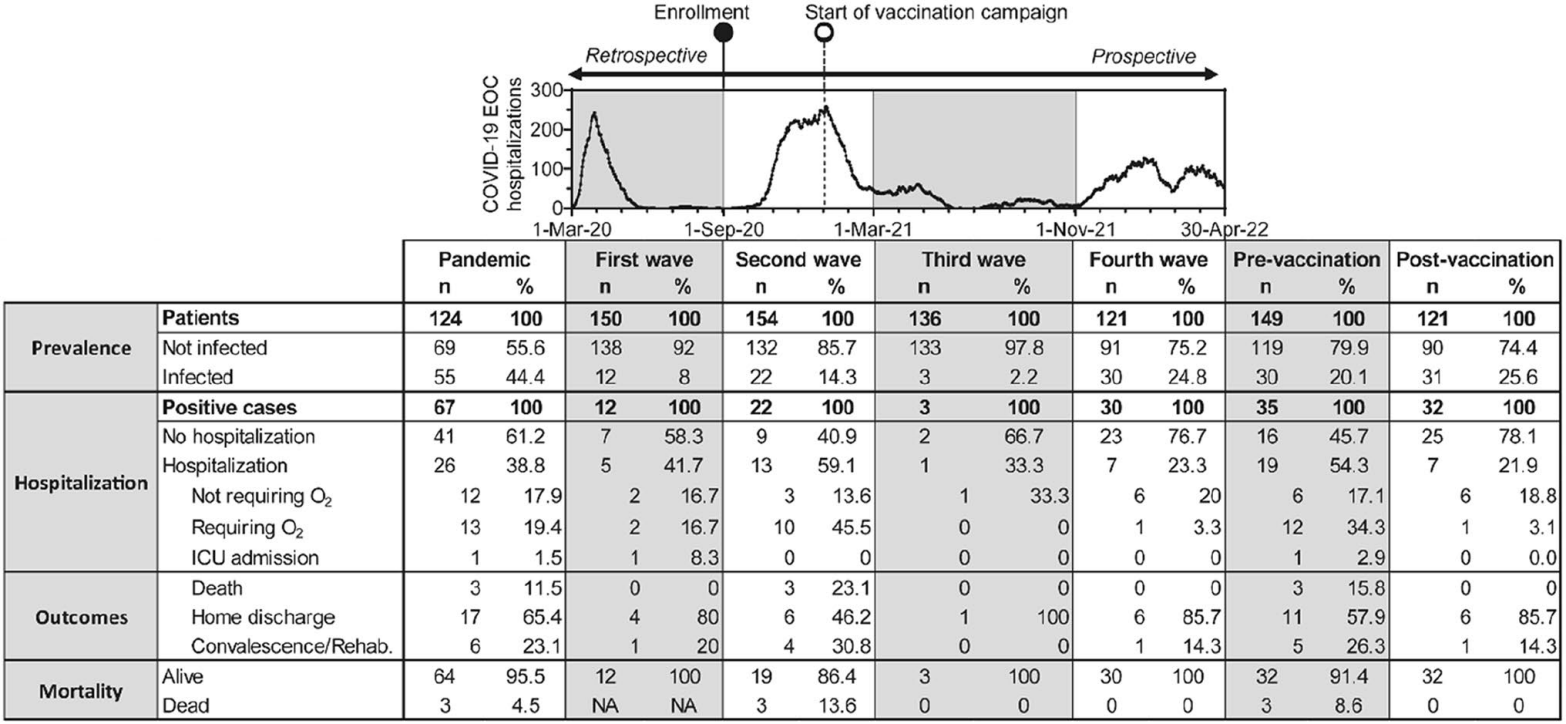
Overview of prevalence and outcomes during the COVID-19 pandemic in dialysis patients. Shown are number (*n*) and percentage (%) of patients and positive cases on maintenance dialysis throughout the pandemic (first 26 months), the individual waves identified on COVID-19-related hospitalizations in the EOC hospital and the pre- and post-vaccination periods. *NA* not applicable evaluation of mortality in the retrospective phase of the study (first wave)

As of September 1, 2020, 165 patients (148 on hemodialysis, 17 on peritoneal dialysis) were enrolled and prospectively followed for 19 months. Of those patients, 150 were already on dialysis treatment as of March 1, 2020, which is considered the beginning of the pandemic outbreak in Southern Switzerland, and their data were retrospectively analyzed. None of the patients were treated with either nirmatrelvir/ritonavir or tixagevimab-cilgavimab, while two patients received sotrovimab. During a 26-month observation period covering the first four pandemic waves, 67 SARS-CoV-2-positive cases were identified by reverse transcriptase-polymerase chain reaction (RT-PCR) and serology among 61 patients (56 were infected once, four twice, and one patient was infected three times). The overall prevalence of SARS-CoV-2 infections among 124 patients on dialysis throughout the four pandemic waves was 44.4%, resulting in an average annual prevalence of 20.5%. Eight percent of patients were infected in the first wave, 14.3% in the second, 2.2% in the third, and 24.8% in the fourth wave. Infection rate in the 112 hemodialysis patients was similar to that of the 12 peritoneal dialysis patients (44.6% vs. 41.7%; *p* = 0.8). With the exception of antiplatelet therapy (OR = 2.11, *p* = 0.047), no other sociodemographic or clinical features were found as risk factors for SARS-CoV-2 infection.

A large number of patients (96.6%, *n* = 144/149) adhered to the COVID-19 vaccination campaign, which started in January 2021. Prevalence of SARS-CoV-2 infection did not significantly change from the pre-vaccination phase (20.1%, *n* = 30/149) to the post-vaccination phase (25.6%, *n* = 31/121, *p* = 0.28). Compared to the pre-vaccination period, the post-vaccination was characterized by a lower hospitalization rate (21.9% vs 54.3%; *p* = 0.007), a shorter length of stay (average 7.3 vs 17.6 days; range 2–46 vs 4–16; *p* = 0.06), a lower rate of patients requiring oxygen (3.1% vs 37.1%; *p* = 0.001), a lower rate of admission to an intensive care unit (ICU; 0% vs 2.9%; *p* = 0.34) and lower mortality related to COVID-19 (0% vs 8.6%; *p* = 0.24).

To the best of our knowledge, this is the first analysis of prevalence of SARS-CoV-2 infection in dialysis patients that considers a more than two-year-long pandemic period. The different infection rates observed during the four waves parallel the transmissibility of the different SARS-CoV-2 variants, with the highest prevalence observed while Delta and Omicron lineages were circulating [1]. Prevalence varied among different studies and countries with values ranging from 0% to 37.6% and with most authors assuming that infection rate in dialysis patients could be considerably higher than that expected in the general population, although comparison data between the two populations in the same region is limited [2]. A seroprevalence study was conducted by the Regional Health Department on a representative cohort of the general population of Canton Ticino estimating a cumulative SARS-CoV-2 infection rate of 22.3% in May 2021 [3]. This rate was comparable to that calculated in our cohort in the same period (19.7%) and to the average annual estimated seroprevalence (20.5%), although dialysis patients were expected to have a higher infection risk due to their vulnerability and to the frequent weekly accesses to the hospital. Instead, our data supports the hypothesis that most infections were acquired outside the hospital, likely due to systematic screenings with triage of symptoms and temperature, frequent PCR-testing even of subjects with mild symptoms leading to rapid identification and isolation of suspected cases, and strict application of distancing, hygiene, and protection protocols, which helped reduce the difference in SARS-CoV-2 infection rates between the dialysis and the general population. Notably, peritoneal dialysis patients, who could be considered more protected because of their home-based treatment, showed an infection rate similar to that of hemodialysis patients.

Despite the almost total adherence to vaccination, no reduction of SARS-CoV-2 infections was observed in the post-vaccination period, as instead described by other studies [4]. Indeed, prevalence of SARS-CoV-2 infection in our cohort was slightly higher (+ 5.5%) after vaccination, consistent with the higher transmissibility of the Omicron variant. The availability of mRNA COVID-19 vaccines has largely reduced the rate of severe courses with, in particular, a lower rate of hospital admission and mortality when compared to unvaccinated patients [4, 5]. This favorable outcome was also observed in our cohort, which showed after vaccination a marked reduction in hospitalization rate (− 32.4%), length of stay (− 10.0 days), respiratory insufficiency requiring oxygen (− 34%), ICU admission (− 2.9% reaching 0%), and COVID-19-related mortality (− 8.6% reaching 0%).

Limitations of the study include the fact that it is single-center and that the sample size is small in a peculiar healthcare context where dialysis patients are carefully attended to thanks to substantial healthcare resources. This might have led to a selection bias for which the small-size sample of our cohort might not be representative of the entire target population. Mortality and outcomes are potentially biased by the selection of patients for this study, which excluded those with low life expectancy. The main strength of this study is the serological and clinical surveillance of the patients over more than two years of the pandemic, with precise medical records permitting to define the cause of death, which allowed us to accurately analyze prevalence, outcomes, and effect of vaccination within each pandemic wave and the pre- and post-vaccination periods.

In conclusion, the high prevalence of SARS-CoV-2 infection in dialysis patients did not differ from that of the general population of Southern Switzerland. Although COVID-19 vaccines did not reduce the prevalence of SARS-CoV-2 infection, they largely decreased the rate of severe outcomes in our dialysis cohort. However, considering that the different variants do not have the same severity, and that patients were differently managed in the various waves, the decrease in hospitalization rate, length of stay, admission to ICU, and mortality can not be attributed solely to vaccination.

### Supplementary Information

Below is the link to the electronic supplementary material.

Supplementary file1 (DOCX 443 KB)

## Data Availability

The data underlying this article will be shared on reasonable request to the corresponding author.
