# Supplementary material for: Prevalence of SARS-CoV-2 infection and impact of vaccination in dialysis patients over two years of the pandemic
Source: J Nephrol. 2023 Sep 13;36(9):2665–7. doi: 10.1007/s40620-023-01754-1 (PMC10703959; doi:10.1007/s40620-023-01754-1)

**Prevalence of SARS-CoV-2 infection and impact of vaccination in dialysis patients over two years of pandemic**

Paolo Hitz, Alberto Pagnamenta, Laura Pertusini, Tatiana Terrot, Yves Franzosi, Jessica Bassi, Chiara Silacci-Fregni, Valeria Gaia, Gladys Martinetti, Franco Keller, Lorenzo Berwert, Valentina Forni Ogna, Soraya Lavorato-Hadjeres, Davide Giunzioni, Andrea D’Ermo, Alan Valnegri, Paolo Ferrari, Davide Corti, Alessandro Ceschi, Pietro Cippà, Luca Piccoli, and Olivier Giannini

**Supplementary Information**

**Table of Contents**

Material and Methods

Supplementary Results

Supplementary References

Supplementary Table 1

Supplementary Table 2

Supplementary Table 3

Supplementary Figure 1

**Materials and Methods**

*Study participants and ethics statement*

The prospective observational analysis included incident hemodialysis (HD) and peritoneal dialysis (PD) patients as of September 1, 2020, treated in one of the four nephrology services of the public multisite hospital of the Ente Ospedaliero Cantonale (EOC) in Ticino region (Southern Switzerland). Participants were excluded if they were under 18 years, with life expectancy lower than 3 months, with severe dementia (CDR ≥ 1) or were unable to understand and give informed consent. Patients were dropped out of the study if they were transferred to a dialysis facility external to EOC, deceased, transplanted or they stopped dialysis treatment. Patients’ clinical course was documented prospectively from September 1, 2020, until April 30, 2022. A retrospective analysis was performed on the enrolled patients who were on dialysis as of March 1, 2020, which is considered the start of the COVID-19 pandemic in Southern Switzerland^1^. The study protocol was approved by the local Institutional Review Boards (Canton Ticino Ethics Committee, Switzerland).

*Laboratory testing*

The definition of a COVID-19-positive case was based on a positive reverse transcriptase-polymerase chain reaction (RT-PCR) test on samples obtained by nasopharyngeal swab and/or by a positive serological test (anti-SARS-CoV-2 S IgG ELISA) performed on blood samples collected before vaccination. The nasopharyngeal swab was performed or requested by the nephrologist of the patients and all positive tests were reported by a twice-weekly report of the EOC central laboratory to the principal investigator of the study. Plasma was isolated from blood draw performed using BD tubes containing Ficoll (BD, CPT Ficoll, Cat. No. 362780) and stored at +4°C. ELISA was performed and ED50 titers were determined as previously described^2^. Positive samples were defined by ED50>50.

*Data acquisition*

Demographic, clinical and outcome data were collected at 6 different timepoints during the 25 month of the study period. Electronic case report forms (eCRFs) were used to capture all project-related data. Once the eCRFs were fully completed, all data were reviewed to ensure the accuracy, completeness and clarity of the reported information. Infection prevalence, outcome data and mortality rate of patients hospitalized in the EOC Hospital network were collected from the *EOC Covid – 19 dashboard*, a database created in March 2020 to monitor the COVID-19 health emergency in our region including information such as laboratory analyses, deaths, occupation of intensive care beds, vaccination status, distribution by age group and use of emergency services.

*Definition of the pandemic waves, pre-/post-vaccination period and underlying cause of death (UCOD)*

Since there is still no consensus on the definition of a wave during a pandemic and the evolution of each wave depends on the region, we considered the start of a new wave when a rapid raise of incident infection cases was described after a period of stable or declining cases. For this purpose, we used the table and statistics of SARS-CoV-2 infection data from the internal *COVID-19 dashboard* of EOC and from the epidemiological data registry of the Regional Health Department (DSS) of Ticino region^1^. The first pandemic wave was defined from March 1 to August 31, 2020; the second wave from September 1, 2020, to February 28, 2021; the third wave from March 1 to October 31, 2021; the fourth wave from November 1, 2021, to April 30, 2022.

Since dialysis patients were individually vaccinated with a COVID-19 mRNA vaccine between January 1 and March 31, 2021, we defined the pre- and post-vaccination period individually.

According to the CDC clinical guidance^3^ and the criteria proposed by the local health authorities^1^, COVID–19 was defined as the underlying cause of death (UCOD) when a proven SARS-CoV-2 infection causing life-threatening conditions, such as respiratory insufficiency, pneumonia, and/or an acute respiratory distress syndrome, was described in the patient’s medical report.

*Statistical analysis*

Descriptive quantitative patients’ characteristics were presented as mean with standard deviation or as median with 25^th^ and 75^th^ percentile, as appropriate. Quantitative data were summarized with absolute numbers and corresponding percentages. Comparisons of the different variables between groups were performed with parametric two-sample t test and non-parametric Chi-square or two-sample Wilcoxon rank-sum/Mann-Whitney U test, as appropriate. Potential predictors of the occurrence of infection were first selected by univariate logistic regression analysis. Predictors with a *p*-value < 0.2 were then entered in a multivariable logistic regression model. All tests were performed two-sided and p-value < 0.05 was considered statistically significant. All statistical analysis were performed using Stata version 15 software (StataCorp LP, College Station, TX, USA).

**Supplementary Results**

*Baseline characteristics*

As of September 1, 2020, 221 patients were on maintenance HD (n= 198) or PD (n= 23) treatment in one of the four nephrology services of the EOC hospital. With a 74.4% adherence, 165 patients (148 HD, 17 PD) were enrolled according to the inclusion and exclusion criteria of the study and were prospectively followed for 19 months. As of April 30, 2022, 121 patients were still enrolled at the end of the study, with 13 dropped out due to transplantation, 2 due to dialysis interruption, 2 due to transfer to a different center and 27 patients died. The median age of the enrolled patients was 75 years (25^th^-75^th^ percentile 66-83) and 65% of them were male. Hypertension (84%) and cardiovascular disease (50%) were the most common comorbidities followed by diabetes (40%). Among the 144 patients vaccinated for COVID-19, 83% received the BNT162b2 SARS-CoV-2 mRNA vaccine from Pfizer-BioNTech and 17% received the mRNA-1273 vaccine from Moderna. **Supplementary Table 1** shows a summary of the patients’ demographics, dialysis characteristics and comorbidities.

*Identification of SARS-CoV-2 positive cases*

Patients with COVID-19-related symptoms were tested by RT-PCR, which confirmed 60 positive cases of SARS-CoV-2 infection during the whole observation period (28 in the pre-vaccination and 32 in the post-vaccination period). Serological analysis of samples collected after infection and before vaccination confirmed the presence of anti-SARS-CoV-2 S IgG in 17 of the 28 PCR-positive cases and identified 7 additional asymptomatic cases, who had never been tested by RT-PCR. Prevalence during the pandemic period was calculated from 55 positive cases out of 124 patients, which include 121 patients still enrolled at the end of the study and 3 patients dropped out due to COVID-19-related death (**Figure 1** and **Supplementary Figure 1a**).

*Comparison of the COVID-19-positive with the COVID-19-negative dialysis patients*

61 out of the 165 patients (37%) were COVID-19-positive during the prospective follow-up of the study. When age, sex, household situation, dialysis modality, comorbidities, seasonal influenza vaccination and pharmacological treatments were compared in COVID-19-positive and negative patients, none of these variables was independently found as a risk factor for SARS-CoV-2 infection (**Supplementary Table 2**), with the exception of antiplatelet therapy that was significant in a multivariable analysis (OR = 2.11, p = 0.047, **Supplementary Table 3**).

*Outcomes in dialysis patients during the four pandemic waves*

20 patients out of the 67 infection cases (29.8%) were admitted to the hospital because of COVID-19 and additional 6 patients (9%) had a diagnosis of SARS-CoV-2 infection during hospitalization for other causes without a COVID-19 illness. Of the 26 COVID-19 patients, 14 (53.8%) had respiratory insufficiency requiring oxygen but only one patient (3.8%) was admitted to an intensive care unit (ICU) (**Supplementary Figure 1b**). The average hospital length of stay (LOS) of all 26 infected cases was 14.8 days (median 12.5, range 2-46), which was comparable to that of the overall patients hospitalized with a SARS-CoV-2 infection in the EOC hospital (average 12.7, median 9, range 5-16, p= 0.28) in the same period. The average LOS was longer during the first and the second wave (23.2 and 16.5 days, average 18.4) compared to the third and the fourth wave (7.0 and 6.7 days, average 6.8, p= 0.016). Pharmacological treatments mainly included high-dose glucocorticoids in 5/34 patients (14.7%) during the first two waves and in 1/33 patients (3%) during the next two waves, as well as sotrovimab^4^, a human neutralizing monoclonal antibody, which was administered to 2 out of 30 patients only during the fourth wave (6.6%). Most of the 26 hospitalized patients were discharged at home (n= 17, 65.4%), others transferred to a rehabilitation or convalescence facility (n= 6, 23.1%), while 3 patients (11.5%) died with a SARS-CoV-2 infection during the second wave (**Supplementary Figure 1c**). These three deaths represent an infection-fatality ratio of 4.9% of the 61 COVID-19 patients. However, in only one case COVID-19 was identified as the underlying cause of death (UCOD) resulting in a COVID-19-associated mortality of 1.6% of the 61 infected patients (**Supplementary Figure 1d**).

**Supplementary References**

**1.** Ticino ReC. Coronavirus. 2022. <https://www4.ti.ch/dss/dsp/covid19/home>, 2022.

**2.** Bassi J, Giannini O, Silacci-Fregni C, et al. Poor neutralization and rapid decay of antibodies to SARS-CoV-2 variants in vaccinated dialysis patients. *PLoS One* 2022;17:e0263328.

**3.** Prevention CfDCa. COVID-19. 2022. <https://www.cdc.gov/coronavirus/2019-ncov/>.

**4.** Heo YA. Sotrovimab: First Approval. *Drugs* 2022;82:477-484.

| **Supplementary Table 1. Socio-demographic and clinical data of the dialysis cohort analyzed** | |
| --- | --- |
| **Enrolled patients, n (%)** | 165 (100%) |
| **Age, min-max, median, 25^th^-75^th^ percentile, years** | 25-97, 75, 66-83 |
| **Sex, female, n (%)** | 58 (35%) |
| **BMI, min-max, median, 25^th^-75^th^ percentile, kg/m^2^** | 15.6-58.8, 25.7, 22.8-29.2 |
| **Smoker, n (%)** | 28 (17%) |
| **Household situation, n (%)** |  |
| **Living alone** | 47 (28%) |
| **Living in the family** | 106 (64%) |
| **Living in a medical institution** | 11 (7%) |
| **Other** | 1 (1%) |
| **Transfer to dialysis, n (%)** |  |
| **Private alone** | 70 (42%) |
| **Private, not alone** | 35 (21%) |
| **Organized transfer** | 59 (36%) |
| **Public transportation** | 1 (1%) |
| **Dialysis treatment, n (%)** |  |
| **HD** | 48 (29%) |
| **HDF** | 100 (61%) |
| **PD** | 17 (10%) |
| **Dialysis vintage, min-max, median,**  **25^th^-75^th^ percentile, months** | 11-268, 43, 27-80 |
| **Dialysis access, native AVF, n (%)** | 106 (64%) |
| **Renal Disease, n (%)** |  |
| **Primary glomerulonephritis** | 22 (13%) |
| **Secondary glomerulonephritis** | 19 (12%) |
| **Chronic pyelonephritis** | 6 (4%) |
| **Interstitial disease** | 5 (3%) |
| **Genetic disease** | 1 (1%) |
| **Polycystic kidney disease** | 12 (7%) |
| **Renovascular disease** | 61 (37%) |
| **Diabetic glomerulopathy** | 29 (18%) |
| **Other** | 10 (6%) |
| **Comorbidities, n (%)** |  |
| **Obesity** | 28 (17%) |
| **Diabetes** | 66 (40%) |
| **Hypertension** | 138 (84%) |
| **Heart failure** | 25 (15%) |
| **Cardiovascular disease** | 83 (50%) |
| **Pulmonary disease** | 51 (31%) |
| **Chronic liver disease** | 15 (9%) |
| **Gastrointestinal disease** | 33 (20%) |
| **Haemato-oncological disease** | 15 (9%) |
| **Autoimmune disease** | 13 (8%) |
| **Kidney transplant** | 10 (6%) |
| **Neurologic disease** | 5 (3%) |
| **Positive HbS antigen, n (%)** | 2 (1%) |
| **Positive HbS antibodies, n (%)** | 124 (75%) |
| **Positive HCV antibodies, n (%)** | 24 (15%) |
| **HIV positive, n (%)** | 0 (0%) |
| **2020 seasonal flu vaccination, n (%)** | 113 (68%) |
| **Pharmacological treatment, n (%)** |  |
| **Oral anticoagulation** | 29 (18%) |
| **Antiplatelet drugs** | 103 (62%) |
| **ACE inhibitors/ARBs** | 79 (48%) |
| **Calcium antagonists** | 68 (41%) |
| **Beta blockers** | 101 (61%) |
| **Statins** | 90 (55%) |
| **Oral antidiabetic drugs** | 27 (16%) |
| **Insulin** | 40 (24%) |
| **Immunosuppressive drugs^§^** | 13 (8%) |
| **Oral/intravenous steroids** | 19 (12%) |
| **Vitamin D derivatives** | 127 (77%) |
| BMI, body mass index. HDF, hemodiafiltration. AVF, arteriovenous fistula. ACE, angiotensin-converting enzyme. ARB, angiotensin receptor blockers. ^§^includes any of the following: tacrolimus, sirolimus, everolimus, cyclosporine, cyclophosphamide, mycophenolate mofetil, azathioprine, rituximab | |

**Supplementary Table 2. Comparison of 61 COVID-19-positive and 104 negative dialysis patients (univariate logistic regression)**

| **Variable** | **OR** | **95%-CI** | **P-value** |
| --- | --- | --- | --- |
| **Age** | 0.98 | 0.96-1.01 | 0.181 |
| **Sex (female as reference)** | 0.75 | 0.39-1.48 | 0.410 |
| **BMI** | 1.00 | 0.94-1.06 | 0.998 |
| **Smoke** | 0.99 | 0.69-1.44 | 0.987 |
| **Household situation** | 1.32 | 0.76-2.29 | 0.321 |
| **Transfer to dialysis** | 0.91 | 0.72-1.16 | 0.459 |
| **Dialysis treatment** | 1.20 | 0.70-2.03 | 0.508 |
| **Dialysis vintage** | 1.00 | 0.99-1.00 | 0.432 |
| **Comorbidities** |  |  |  |
| **Diabetes mellitus** | 0.96 | 0.52-1.83 | 0.895 |
| **Hypertension** | 1.48 | 0.61-3.620 | 0.390 |
| **Heart failure** | 0.77 | 0.31-1.91 | 0.577 |
| **Cardiovascular disease** | 1.53 | 0.79-2.97 | 0.211 |
| **Pulmonary disease** | 0.62 | 0.30-1.25 | 0.180 |
| **Chronic liver disease** | 1.55 | 0.53-4.52 | 1.555 |
| **Gastrointestinal disease** | 1.33 | 0.61-2.90 | 0.469 |
| **Haemato-oncological disease** | 1.15 | 0.39-3.41 | 0.799 |
| **Autoimmune disease** | 0.74 | 0.22-2.52 | 0.630 |
| **Kidney transplant** | 1.15 | 0.31-4.23 | 0.838 |
| **Neurologic disease** | 1.14 | 0.19-7.03 | 0.887 |
| **Positive HbS antigen** | 1.71 | 0.11-27.95 | 0.704 |
| **Positive HbS antibodies** | 1.06 | 0.51-2.18 | 0.878 |
| **Positive HCV antibodies** | 0.77 | 0.25-2.41 | 0.656 |
| **2020 seasonal flu vaccination** | 0.98 | 0.95-1.00 | 0.624 |
| **Pharmacological treatment** |  |  |  |
| **Oral anticoagulation** | 0.73 | 0.31-1.72 | 0.467 |
| **Antiplatelet drugs** | 1.55 | 0.80-3.03 | 0.193 |
| **ACE inhibitors** | 1.49 | 0.66-3.36 | 0.336 |
| **Angiotensin receptor blockers** | 0.83 | 0.41-1.67 | 0.603 |
| **Calcium antagonists** | 1.22 | 0.64-2.31 | 0.542 |
| **Beta blockers** | 0.62 | 0.33-1.19 | 0.152 |
| **Statins** | 0.97 | 0.51-1.83 | 0.930 |
| **Oral antidiabetic drugs** | 1.45 | 0.63-3.35 | 0.381 |
| **Insulin** | 1.56 | 0.76-3.22 | 0.229 |
| **Rituximab** | 1.71 | 0.11-27.95 | 0.704 |
| **Tacrolimus** | 0.85 | 0.08-9.57 | 0.895 |
| **Cyclosporin** | 0.42 | 0.05-3.82 | 0.438 |
| **Oral steroids** | 1.10 | 0.40-2.99 | 0.858 |
| **Intravenous steroids** | 1.72 | 0.11-27.95 | 0.704 |
| **Vitamin D derivatives** | 1.16 | 0.55-2.50 | 0.688 |

**Supplementary Table 3. Comparison of 61 COVID-19-positive and 104 negative dialysis patients (univariate and multivariate logistic regression)**

|  | **Univariate logistic regression** | | | **Multivariable logistic regression** | | |
| --- | --- | --- | --- | --- | --- | --- |
| **Variable** | **OR** | **95%-CI** | **P-value** | **OR** | **95%-CI** | **P-value** |
| **Age** | 0.98 | 0.96-1.01 | 0.181 | 0.98 | 0.95-1.00 | 0.106 |
| **Sex (female as reference)** | 0.75 | 0.39-1.48 | 0.410 | 0.76 | 0.37-1.53 | 0.435 |
| **Pulmonary disease (no as reference)** | 0.62 | 0.30-1.25 | 0.180 | 0.55 | 0.26-1.16 | 0.115 |
| **Antiplatelet drugs (no as reference)** | 1.55 | 0.80-3.03 | 0.193 | 2.11 | 1.01-4.40 | 0.047 |
| **Beta blockers (no as reference)** | 0.62 | 0.33-1.19 | 0.152 | 0.52 | 0.26-1.02 | 0.057 |

**Supplementary Figure 1. Prevalence (a), hospitalization (b), outcomes (c) and mortality (d) of SARS-CoV-2 infection in dialysis patients during pandemic and comparison between waves and pre- and post-vaccination period.**


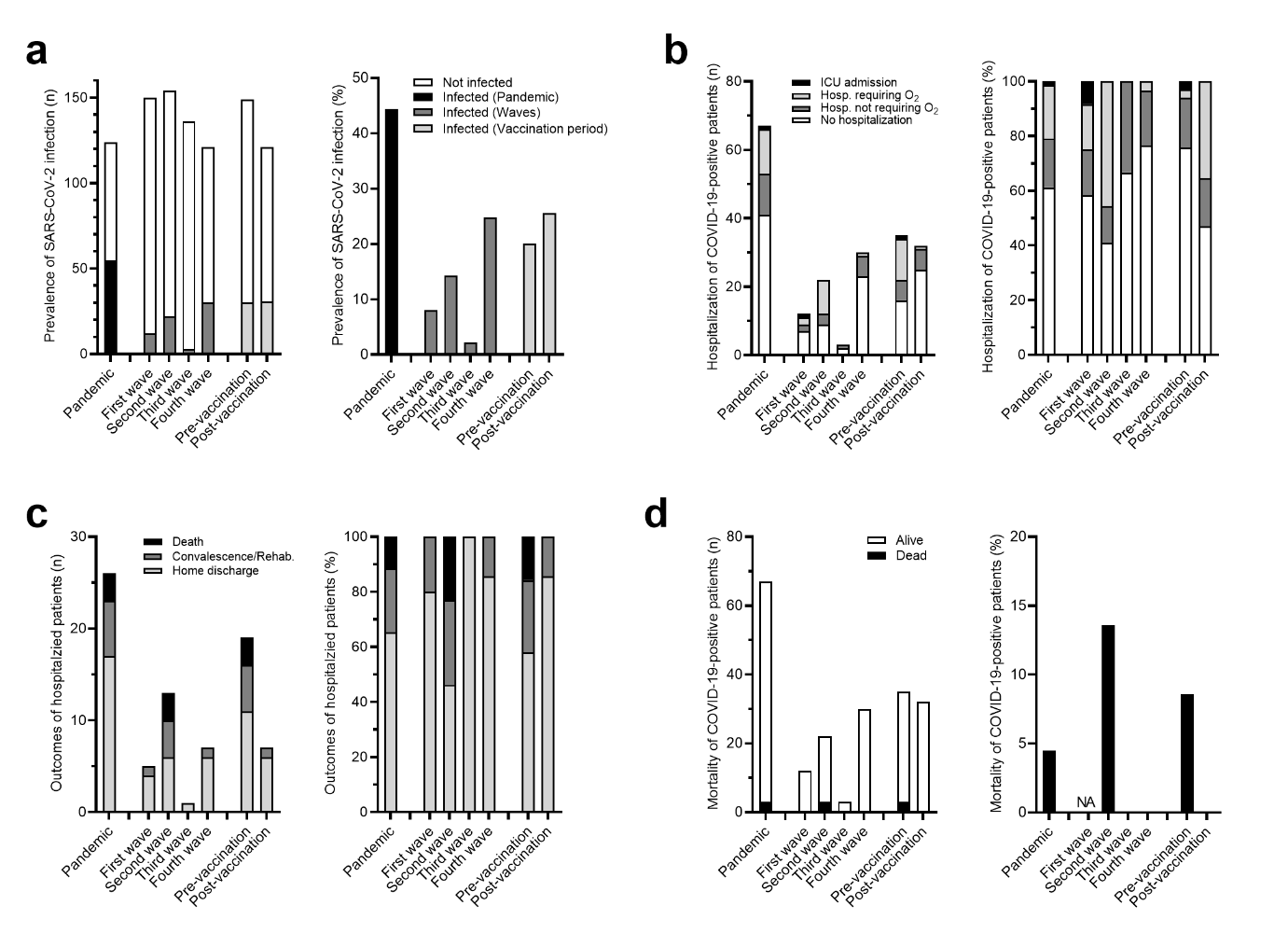

Supplement: Supplementary file 1 — Supplementary file1 (DOCX 443 KB) [file 40620_2023_1754_MOESM1_ESM.docx]
